# WISP-1 positively regulates angiogenesis by controlling VEGF-A expression in human osteosarcoma

**DOI:** 10.1038/cddis.2016.421

**Published:** 2017-04-13

**Authors:** Hsiao-Chi Tsai, Huey-En Tzeng, Chun-Yin Huang, Yuan-Li Huang, Chun-Hao Tsai, Shih-Wei Wang, Po-Chuan Wang, An-Chen Chang, Yi-Chin Fong, Chih-Hsin Tang

**Affiliations:** 1 Graduate Institute of Basic Medical Science, China Medical University, Taichung, Taiwan; 2 Division of Hematology/Oncology, Department of Medicine, Taichung Veterans General Hospital, Taichung, Taiwan; 3 School of Medicine, China Medical University, Taichung, Taiwan; 4 Department of Orthopaedic Surgery, China Medical University Beigang Hospital, Yun-Lin County, Taiwan; 5 Department of Biotechnology, College of Health Science, Asia University, Taichung, Taiwan; 6 Department of Orthopedic Surgery, China Medical University Hospital, Taichung, Taiwan; 7 Department of Medicine, Mackay Medical College, New Taipei City, Taiwan; 8 Department of Gastroenterology, Hsinchu MacKay Memorial Hospital, Hsinchu City, Taiwan; 9 Institute of Biomedical Sciences, National Chung Hsing University, Taichung, Taiwan; 10 Department of Sports Medicine, College of Health Care, China Medical University, Taichung, Taiwan

## Abstract

In recent years, much research has focused on the role of angiogenesis in osteosarcoma, which occurs predominantly in adolescents and young adults. The vascular endothelial growth factor-A (VEGF-A) pathway is the key regulator of angiogenesis and in osteosarcoma. VEGF-A expression has been recognized as a prognostic marker in angiogenesis. Aberrant WNT1-inducible signaling pathway protein-1 (WISP-1) expression is associated with various cancers. However, the function of WISP-1 in osteosarcoma angiogenesis is poorly understood. We demonstrate a positive correlation between WISP-1 and VEGF-A expression in human osteosarcoma. Moreover, we show that WISP-1 promotes VEGF-A expression in human osteosarcoma cells, subsequently inducing human endothelial progenitor cell (EPC) migration and tube formation. The focal adhesion kinase (FAK), Jun amino-terminal kinase (JNK), and hypoxia-inducible factor (HIF)-1*α* signaling pathways were activated after WISP-1 stimulation, while FAK, JNK, and HIF-1*α* inhibitors or small interfering RNA (siRNA) abolished WISP-1-induced VEGF-A expression and angiogenesis. *In vitro* and *in vivo* studies revealed down-regulation of microRNA-381 (miR-381) in WISP-1-induced VEGF-A expression and angiogenesis. Our findings reveal that WISP-1 enhances VEGF-A expression and angiogenesis through the FAK/JNK/HIF-1*α* signaling pathways, as well as via down-regulation of miR-381 expression. WISP-1 may be a promising target in osteosarcoma angiogenesis.

## 

Osteosarcoma is the most common type of cancer that arises in bone, commonly in the extremities of long bones near metaphyseal growth plates or around the knee.^1^ In recent years, much research has focused on the role of angiogenesis in osteosarcoma proliferation, migration, and metastasis.^2, 3, 4^ Tumor angiogenesis has been recognized in the imbalance between pro-angiogenic and anti-angiogenic factors.^5^ Vascular endothelial growth factor (VEGF)-A is considered to be a master regulator of angiogenesis.^6, 7^ We have previously reported an association between VEGF-A expression and the clinical stages of osteosarcoma.^8^ Our present study sought to understand the mechanism of VEGF-A imbalance in human osteosarcoma.

WNT1 inducible signaling pathway protein-1 (WISP-1), also known as CCN4 or Elm1, is a cysteine-rich protein that belongs to the CCN family.^9^ WISP-1 is expressed during embryonic development and tissue repair.^10^ Aberrant WISP-1 expression is associated with various pathologies including osteoarthritis, fibrosis, and cancer.^11^ Recent studies have confirmed the functional interaction of WISP-1 with integrins.^12^ Integrin *α*v*β*3 is known to exert both pro- and anti-angiogenic functions.^13^ Previous research has reported overexpression of integrin *α*v*β*3 in 80% of neo-endothelial cells and 20% of tumor cells, and its expression can still be detected after chemotherapy treatment in osteosarcoma.^14^ Integrin *α*v*β*3 has been implicated in the regulation of WISP-1-promoted osteosarcoma metastasis.^15^

MicroRNAs (miRNAs) have emerged as crucial players regulating the magnitude of gene expression in a variety of organisms.^16^ MiRNAs are short (≈22 nucleotides) noncoding RNA molecules, which have been shown to participate in many cellular processes, and their dysregulation is observed in different human pathologies, including cancer.^17^ Previous studies have shown that miRNAs inhibit tumor angiogenesis through the dysregulation of the miRNA/VEGF-A axis.^18^ MiR-381 has been found to be involved in cancer progression, including chondrosarcoma, ovarian cancer, hepatocellular carcinoma, and colorectal cancer.^19, 20, 21, 22^ However, the role of miR-381 in osteosarcoma is poorly understood.

In this study, we investigated the role of WISP-1 in osteosarcoma angiogenesis. We found that WISP-1 enhances VEGF-A expression and tumor angiogenesis through the FAK, JNK, and hypoxia-inducible factor (HIF)-1*α* signaling pathways, as well as via down-regulation of miR-381 expression. We also found differential WISP-1 and VEGF-A expression between tumor and normal tissue, and correlation of its expression with clinical outcome. These findings indicate that WISP-1 is a promising target in the clinical diagnosis and prognosis of cancers.

## Results

### WISP-1 promotes VEGF-A expression and angiogenesis

**Figure 1:**
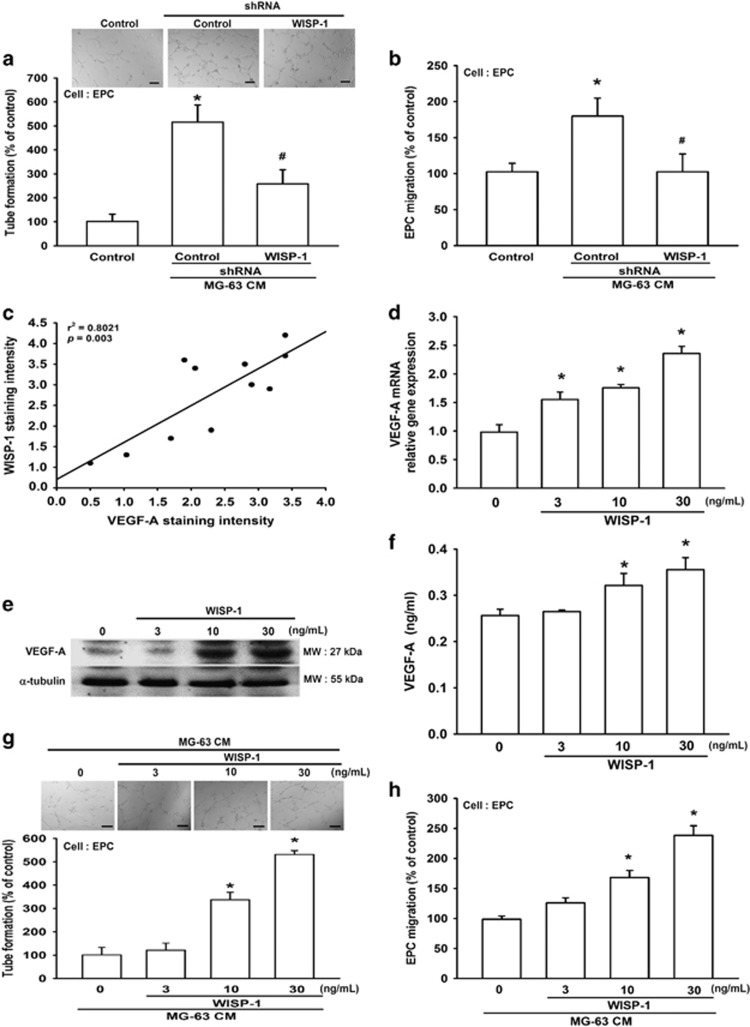
WISP-1 promotes VEGF-A expression and angiogenesis. (**a** and **b**) Cultured medium was collected as CM and then applied to EPCs for 24 h. EPC capillary-like structure formation and cell migration were examined by tube formation (bar=100 *μ*m) and Transwell assay. Uncultured MG-63 medium was used as the control. (**c**) Quantitative results and an observed correlation between WISP-1 and VEGF-A in osteosarcoma patients (*N*=20). (**d**-**f**) After MG-63 cells were treated with various concentrations of WISP-1, mRNA and protein expressions were detected by RT-qPCR, western blot (MW: molecular weight), and enzyme-linked immunosorbent assay. Cells treated with 0 mg/ml of WISP-1 were used as the control. (**g** and **h**) MG-63 cells were treated with various concentrations of WISP-1. Culture medium was collected as CM and applied to the EPCs for 24 h. EPC capillary-like structure formation was examined by tube formation (bar=100 *μ*m) and cell migration by the Transwell migration assay. CM collected from cells treated with 0 mg/ml of WISP-1 was used as the control. Each experiment was performed in triplicate (*N*=3). Results are expressed as the mean±S.E.M. *P<0.05 compared with control; ^#^*P*<0.05 compared with the control-shRNA group

Angiogenesis is required for invasive tumor metastasis and constitutes an important factor in the control of cancer progression.^23^ Our previous investigations found that WISP-1 promotes human osteosarcoma cell migration,^15^ but the effects of WISP-1 on angiogenesis remain largely unknown. Using an *in vitro* tube formation assay, we investigated the effects of WISP-1 on endothelial progenitor cell (EPC) angiogenesis. First, we collected MG-63/control- or MG-63/WISP-1-shRNA culture medium as conditioned media (CM), which was then co-cultured with EPCs. We found that WISP-1 shRNA diminished osteosarcoma CM-enhanced tube formation (Figure 1a). In addition, osteosarcoma CM increased EPC migration, whereas this effect was antagonized with WISP-1 small hairpin RNA (shRNA; Figure 1b). It is known that VEGF-A has a pivotal role in the angiogenesis process.^24^ We reviewed our previous tissue array results^15, 25^ and identified a positive correlation between WISP-1 and VEGF-A expression in osteosarcoma patients (Figure 1c). Next, we directly applied WISP-1 to the human osteosarcoma cell line and examined VEGF-A expression. We found that WISP-1 increased mRNA and protein expression of VEGF-A in a concentration-dependent manner (Figures 1d–f). CM from WISP-1-treated osteosarcoma cells enhanced EPC tube formation and migration in a concentration-dependent manner (Figures 1g–h). These data demonstrate that WISP-1 promotes VEGF-A expression and angiogenesis in human osteosarcoma cells.

### WISP-1 increases VEGF-A expression and angiogenesis through integrin *α*v*β*3

**Figure 2:**
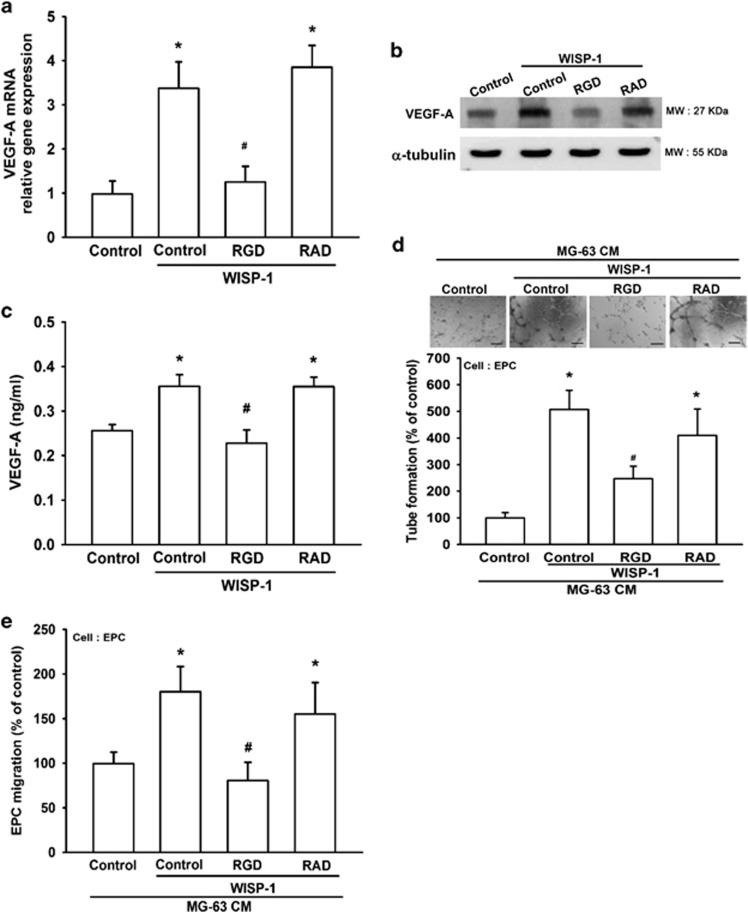
WISP-1 increases VEGF-A expression and angiogenesis via *α*v*β*3 integrin. (**a**–**c**) MG-63 cells were pretreated with RGD (10 *μ*M) or RAD (10 *μ*M) for 30 min, then treated with WISP-1 (30 ng/ml) for 24 h. mRNA and VEGF-A protein expression was detected by RT-qPCR, western blot (MW, molecular weight), and enzyme-linked immunosorbent assay. Untreated cells were used as the control. (**d** and **e**) MG-63 cells were pretreated with RGD (10 *μ*M) or RAD (10 *μ*M) for 30 min, then treated with WISP-1 (30 ng/ml) for 24 h. Culture medium was collected as CM and then applied to the EPCs for 24 h. EPC capillary-like structure formation was examined by tube formation (bar=100 *μ*m) and cell migration by the Transwell migration assay. CM collected from untreated cells was used as the control. Each experiment was performed in triplicate (*N*=3). Results are expressed as the mean±S.E.M. *P<0.05 compared with control; ^#^*P*<0.05 compared with the WISP-1-treated group

CCN family proteins are known to affect cell migration by binding to cell surface integrin receptors.^26^ Our previous research found that WISP-1 increases tumor metastasis and angiogenesis through interaction with a specific receptor integrin *α*v*β*3.^27, 28^ Therefore, we pretreated cells with cyclic Arg-Gly-Asp (RGD; a specific peptide that has high affinity with *α*v*β*3 integrin) or with cyclic Arg-Ala-Asp (RAD) for 30 min and then stimulated cells with WISP-1 for 24 h. We found that treatment of cells with cyclic RGD but not cyclic RAD inhibited WISP-1-induced mRNA and protein expression of VEGF-A (Figures 2a–c). Compared with the control group, cyclic RGD but not cyclic RAD suppressed WISP-1-enhanced EPC tube formation and migration (Figures 2d–e). These findings suggest that WISP-1 induces VEGF-A expression and angiogenesis through the *α*v*β*3 integrin receptor in human osteosarcoma cells.

### The FAK/JNK signaling pathway is involved in WISP-1-induced VEGF-A expression and angiogenesis

**Figure 3:**
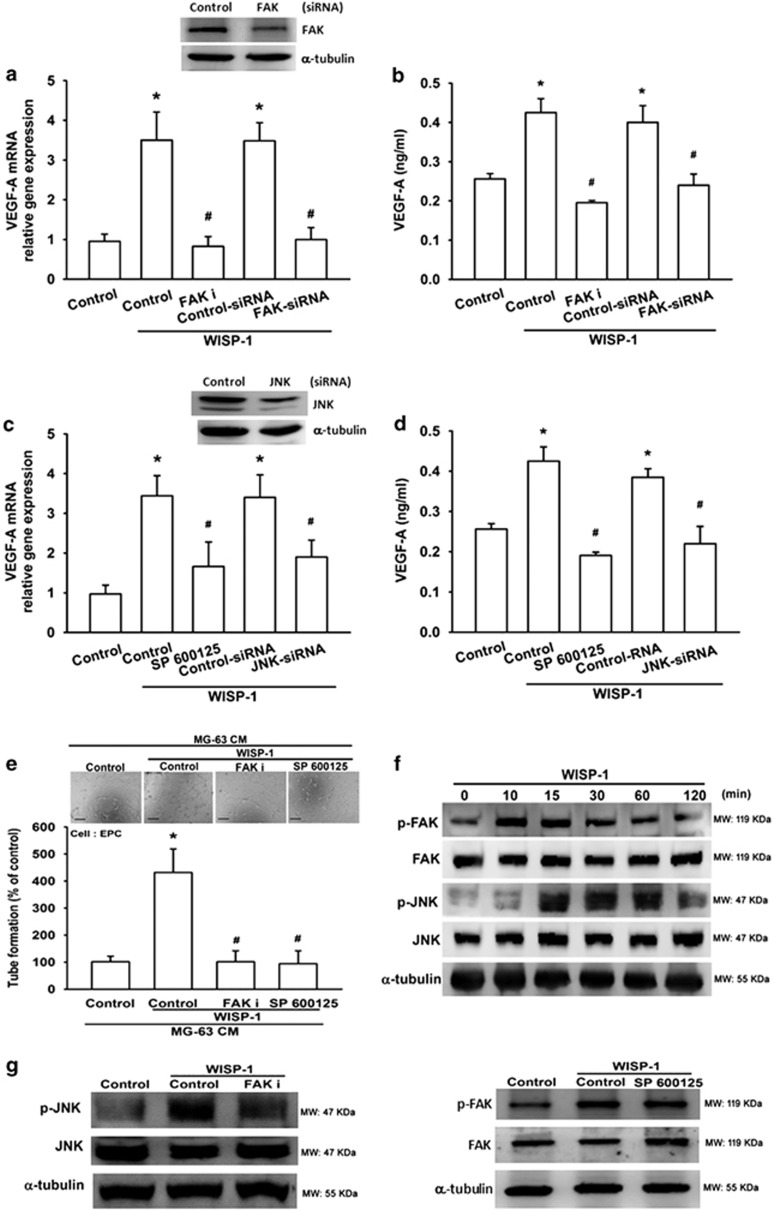
FAK and JNK signaling pathways are involved in WISP-1-induced VEGF-A expression and angiogenesis. (**a** and **b**) MG-63 cells were pretreated with a FAK inhibitor (FAK i; 10 *μ*M) for 30 min or a FAK siRNA for 24 h, before treatment with WISP-1 (30 ng/ml) for 24 h. mRNA was quantified using RT-qPCR and VEGF-A protein expression was assayed by enzyme-linked immunosorbent assay (ELISA). Untreated cells were used as the control. (**c** and **d**) MG-63 cells were pretreated with a JNK inhibitor (SP600125; 10 *μ*M) for 30 min or a JNK siRNA for 24 h, before treatment with WISP-1 (30 ng/ml) for 24 h. mRNA was quantified using RT-qPCR and VEGF-A protein expression was assayed by ELISA. Untreated cells were used as the control. (**e**) MG-63 cells were pretreated with a FAK i or SP600125 for 30 min, then treated with WISP-1 (30 ng/ml) for 24 h. Culture medium was collected as CM and then applied to EPCs for 24 h. EPC capillary-like structure formation was examined by tube formation (bar=100 *μ*m). CM collected from untreated cells was used as the control. (**f**) MG-63 cells were incubated with WISP-1 (30 ng/ml) for the indicated times; FAK and JNK phosphorylation was detected by western blot (MW, molecular weight). (**g**) Cells were pretreated for 30 min with FAK i or SP600125, followed by stimulation with WISP-1 (30 ng/ml). p-FAK and p-JNK expression were detected by western blot. Each experiment was performed in triplicate (*N*=3). Results are expressed as the mean±S.E.M. *P<0.05 compared with control; ^#^*P*<0.05 compared with the WISP-1-treated group

Focal adhesion kinase (FAK) has been implicated as playing an important role in tumor progression, survival, migration, and angiogenesis.^29^ Research has reported that phosphorylated FAK is essential for VEGF-A signaling pathways and angiogenesis.^30^ To verify whether FAK activation is involved in WISP-1-induced VEGF-A expression, we pretreated cells with a FAK inhibitor (FAK i) or FAK siRNA and then stimulated the cells with WISP-1 for 24 h. The results show that FAK siRNA inhibited FAK expression and then diminished WISP-1-enhanced VEGF-A expression (Figures 3a and b). Similarly, WISP-1-enhanced VEGF-A expression was eliminated by the FAK inhibitor (Figures 3a and b). Neither the FAK inhibitor (FAK i) nor the FAK siRNA affected cell integrity or viability, as demonstrated by the immunofluorescence and MTT cell proliferation assays (Supplementary Figure S1). The FAK inhibitor also reduced WISP-1-enhanced EPC tube formation (Figure 3e). In various types of cancers, the phosphorylation of JNK is a downstream molecule of FAK.^31, 32^ We therefore sought to determine whether JNK was involved in WISP-1-induced VEGF-A expression. Pretreatment of cells with JNK siRNA inhibited JNK expression as well as WISP-1-induced VEGF-A expression (Figures 3c and d). The JNK inhibitor (SP600125) abolished WISP-1-induced VEGF-A expression and EPC tube formation (Figures 3c–e). Neither the JNK inhibitor (SP600125) nor the JNK siRNA affected cell integrity or viability, as shown by the immunofluorescence and MTT assays (Supplementary Figure S1). Phosphorylation of FAK and JNK in cells was investigated after WISP-1 stimulation. We found that WISP-1 enhanced FAK and JNK phosphorylation in a time-dependent manner (Figure 3f); maximum phosphorylation values of FAK and JNK were obtained at 10 min and 15 min, respectively. Next, we examined upstream and downstream relationship between FAK and JNK. Pretreatment of cells for 30 min with FAK i decreased JNK phosphorylation (Figure 3g). In contrast, JNK inhibition had no effect on WISP-1-induced FAK phosphorylation (Figure 3g). These results indicate that JNK is a downstream target of FAK. Taken together, our results suggest that WISP-1 induces VEGF-A expression and angiogenesis by activating the FAK/JNK pathway in human osteosarcoma cells.

### Involvement of HIF-1*α* in WISP-1-induced VEGF-A expression and angiogenesis

**Figure 4:**
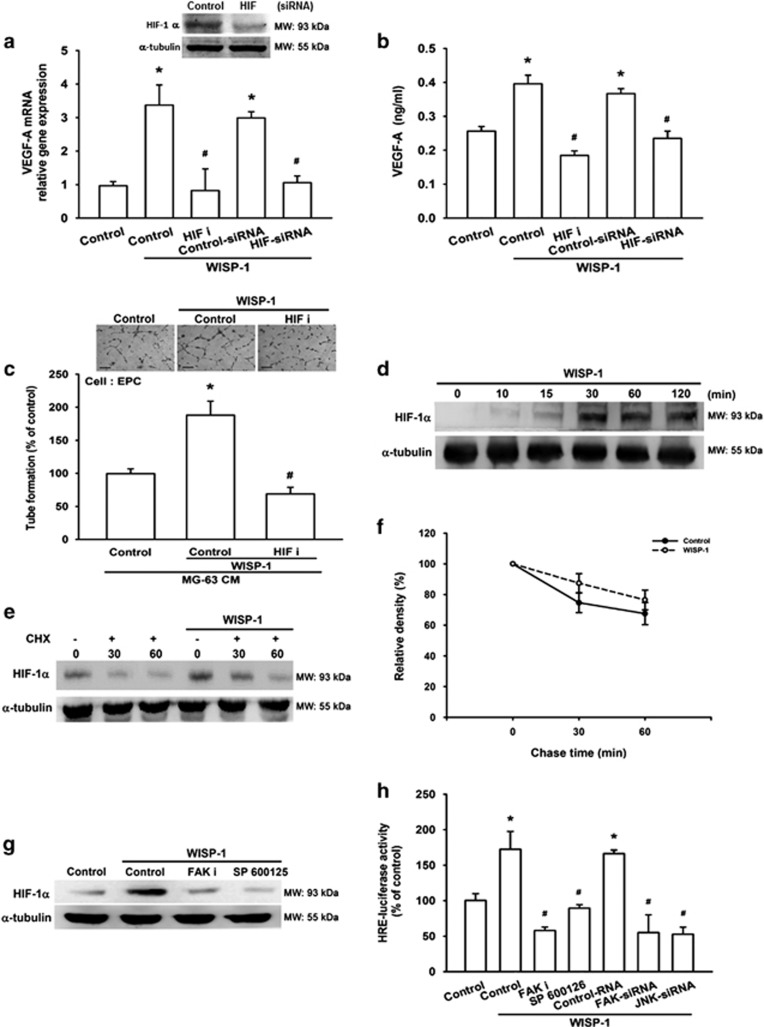
Involvement of HIF-1*α* in WISP-1-induced VEGF-A expression and angiogenesis (**a** and **b**) MG-63 cells were pretreated with an HIF-1 inhibitor (HIF i; 10 *μ*M) for 30 min or HIF-1*α* siRNA for 24 h, then treated with WISP-1 (30 ng/ml) for 24 h. mRNA and VEGF-A protein expression was detected by RT-qPCR and ELISA. Untreated cells were used as the control. (**c**) MG-63 cells were pretreated with an HIF i (10 *μ*M) for 30 min and then treated with WISP-1 (30 ng/ml) for 24 h. Culture medium was collected as CM and then applied to EPCs for 24 h. EPC capillary-like structure formation was examined by tube formation (bar=100 *μ*m). CM collected from untreated cells was used as the control. (**d**) MG-63 cells were incubated with WISP-1 (30 ng/ml) for the indicated times and HIF-1*α* expression was detected by western blot (MW: molecular weight). (**e**) MG-63 cells were treated with WISP-1 (30 ng/ml) for 8 h, then incubated for 0–60 min with CHX 5 *μ*M. HIF-1*α* protein expression was detected by western blot (MW: molecular weight) and the quantification results are shown in (**f**). (**g**) MG-63 cells were pretreated with HIF i or SP600125 for 30 min, then treated with WISP-1 (30 ng/ml) for 24 h. HIF-1*α* protein expression was detected by western blot (MW, molecular weight). Untreated cells were used as the control. (**h**) MG-63 cells were pretreated with HIF i and SP600125 for 30 min or pre-transfected with FAK and JNK siRNA before exposure to WISP-1. HRE-luciferase activity was measured, and the results were normalized to *β*-galactosidase activity. Untreated cells were used as the control. Each experiment was performed in triplicate (*N*=3). Results are expressed as the mean±S.E.M. *P<0.05 compared with control; ^#^*P*<0.05 compared with the WISP-1-treated group

Hypoxia-inducible factor (HIF) has been recognized as an important stimulus for blood vessel growth during tumorigenesis.^6^ HIF-1*α* regulates the expression of a suite of pro-angiogenic genes, including VEGF-A.^33^ We therefore examined whether HIF-1*α* mediates WISP-1-induced VEGF-A expression and angiogenesis. As shown in Figures 4a–c, pretreatment with a HIF-1*α* inhibitor (HIF i) or pre-transfection with a HIF-1*α* siRNA markedly antagonized WISP-1-induced VEGF-A expression and EPC tube formation, but not mRNA levels (Supplementary Figure S2). In contrast, incubation of cells with WISP-1 increased HIF-1*α* protein levels (Figure 4d). Next, we analyzed the possible effect of WISP-1 on HIF-1*α* protein synthesis and performed a time-course analysis of HIF-1*α* turnover in the presence of the protein synthesis inhibitor cycloheximide (CHX). We found that there was no significant change in HIF-1*α* protein half-life with CHX treatment in the presence or absence of WISP-1 (Figures 4e and f), suggesting that WISP-1 enhances HIF-1*α* stability by increasing protein translation, but not by increasing mRNA levels. We further explored whether FAK and JNK signals are involved in WISP-1-induced HIF-1*α* activation. We found that pretreatment with FAK or JNK inhibitors significantly antagonized WISP-1-induced increases in HIF-1*α* expression (Figure 4g). We also used the hypoxia response element (HRE)-luciferase reporter plasmid to examine HIF-1*α* binding ability after WISP-1 stimulation. We found that WISP-1-induced HRE-luciferase activity was significantly reduced by pretreatment with FAK or JNK inhibitors or by pre-transfection with FAK or JNK siRNAs (Figure 4h). Therefore, WISP-1 induces VEGF-A expression and angiogenesis in osteosarcoma through the FAK, JNK and HIF-1*α* pathways.

### WISP-1 promotes VEGF-A expression and angiogenesis by down-regulating miR-381 expression

**Figure 5:**
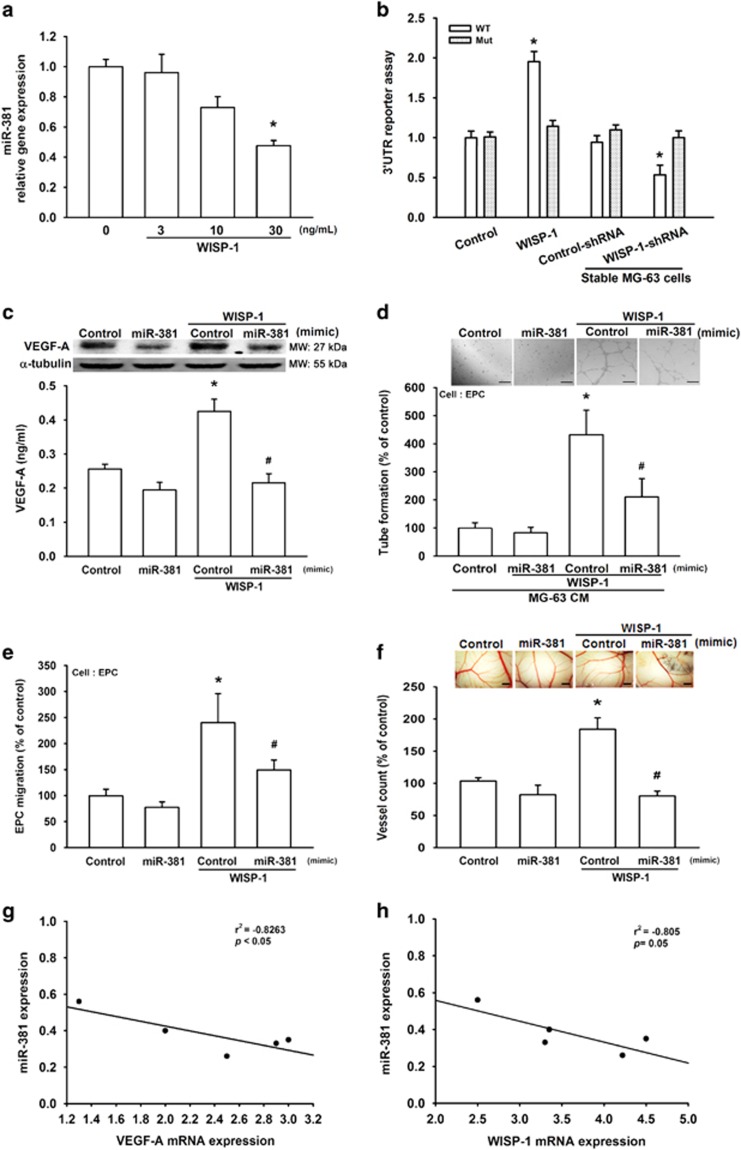
WISP-1 promotes VEGF-A expression and angiogenesis by down-regulating miR-381 expression. (**a**) MG-63 cells were treated with WISP-1 (30 ng/ml) for 24 h, and miR-381 expression was detected by RT-qPCR. Cells treated with 0 mg/ml of WISP-1 were used as the control. (**b**) MG-63 cells (normal and stable) were transfected with 3′UTR reporter assay plasmids for 24 h, then the normal cells were incubated with WISP-1 (30 ng/ml) for 24 h. The relative luciferase activity was measured. Normal cells that were not treated with WISP-1 and the stable control-shRNA cells served as the controls. (**c**) MG-63 cells were transfected with miR-381 mimic for 24 h, then incubated with WISP-1 (30 ng/ml) and VEGF-A expression was detected by western blot (MW, molecular weight) and ELISA. Cells transfected with control miRNA were used as the control. (**d** and **e**) MG-63 cells were transfected with miR-381 mimic for 24 h, then incubated with WISP-1 (30 ng/ml). Culture medium was collected as CM and then applied to the EPCs for 24 h. Capillary-like structure formation and migration in the EPCs was examined by tube formation (bar=100 *μ*m) and the Transwell migration assay, respectively. CM collected from cells transfected with control miRNA was used as the control. (**f**) Cells were transfected with miR-381 mimic for 24 h, then incubated with WISP-1 (30 ng/ml). Culture medium was collected as CM. Chick embryos were incubated with CM for 4 days, then photographed with a stereomicroscope (bar=1 mm) and quantified by vessel count. CM collected from cells that were transfected with control miRNA were used as the control. (**g** and **h**) mRNA expression of WISP-1, VEGF-A, and miR-381 in human osteosarcoma tumor samples was examined by RT-qPCR to determine the correlation between miR-381/VEGF-A and miR-381/WISP-1. Each experiment was performed in triplicate (*N*=3). Results are expressed as the mean±S.E.M. *P<0.05 compared with control; ^#^*P*<0.05 compared with the WISP-1-treated group

Increasing evidence has reported the involvement of miRNAs in the angiogenic process and their potential therapeutic applications for vascular diseases.^17^ We therefore sought to understand which miRNAs are involved in WISP-1-induced VEGF-A expression and angiogenesis. We used open-source software (miRWalk, TargetScan, and microrna.org) to search for possible miRNAs responsible for VEGF-A and ranked the top 14 miRNAs harboring VEGF-A binding sites. After stimulation with WISP-1, we found that miR-381 was the most down-regulated miRNA (Supplementary Table S1). In MG-63 cells, direct application of WISP-1 reduced miR-381 expression in a dose-dependent manner (Figure 5a). To determine whether miR-381 regulates VEGF-A expression by binding to the VEGF-A 3′UTR, we constructed luciferase reporter vectors using the pmiRGLO vector, which harbors the wild-type (pmirGLO-WT; WT) or mutant (pmirGLO-MUT; Mut) 3′UTR of miR-381 (Supplementary Figure S3), and transfected these vectors into MG-63 cells. As shown in Figure 5b, WISP-1 promoted wild-type but not mutant VEGF-A 3′UTR luciferase activity. In addition, knockdown of WISP-1 decreased activity of the wild-type VEGF-A 3′UTR luciferase (Figure 5b). MiR-381 mimic was used to determine the effects of WISP-1-increased VEGF-A expression and angiogenesis. Application of miR-381 mimic reduced WISP-1-induced increases in VEGF-A expression and EPC tube formation, as well as migration (Figures 5c–e). We also used the *in vivo* chick chorioallantoic membrane (CAM) model to detect angiogenesis. We found that the miR-381 mimic diminished WISP-1-enhanced angiogenesis in CAM (Figure 5f). Next, we used clinical osteosarcoma samples to evaluate miR-381 expression. The results indicate that a negative correlation exists between miR-381 and VEGF-A or WISP-1 (Figures 5g–h). It appears that miR-381 has an important role in WISP-1-induced VEGF-A expression and angiogenesis.

### Knockdown of WISP-1 decreases angiogenesis *in vivo*

**Figure 6:**
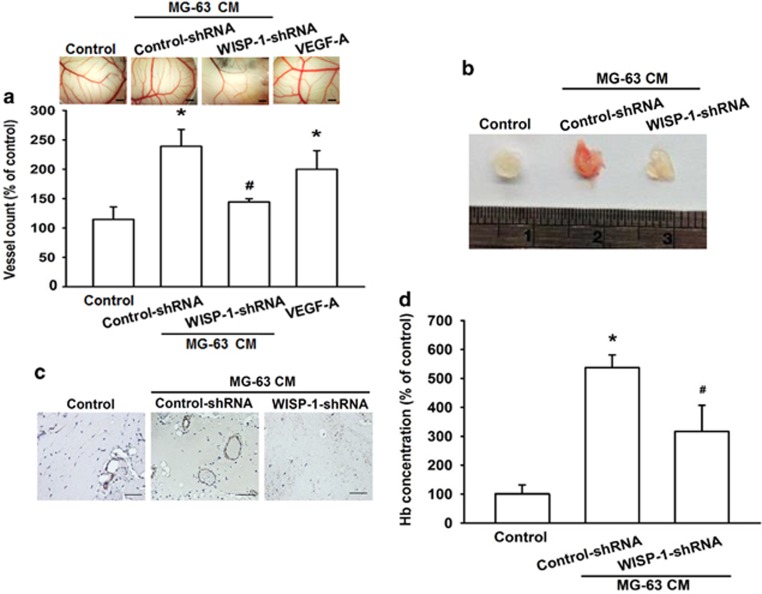
Knockdown of WISP-1 decreases angiogenesis *in vivo*. (**a**) Chick embryos were incubated with osteosarcoma 0% DMEM (control), osteosarcoma CM, or VEGF-A (50 ng/ml) for 4 days, then photographed with a stereomicroscope (bar=1 mm) and quantified by vessel count. (**b**–**d**) Mice were injected subcutaneously with Matrigel mixed with 0% DMEM (control) or osteosarcoma CM for 7 days, then the plugs were excised, photographed, stained with CD31 (bar=50 μm) and quantified for hemoglobin content. Each experiment was performed in triplicate (*N*=3). Results are expressed as the mean±S.E.M. *P<0.05 compared with control; ^#^*P*<0.05 compared with the control-shRNA group

WISP-1-mediated angiogenesis was further demonstrated by the *in vivo* CAM assay. We found that CM from the WISP-1-shRNA group decreased angiogenesis in the CAM model (using VEGF-A as a positive control; Figure 6a). We used the Matrigel plug assay to confirm the results from the CAM assay. An analysis of CD31 staining revealed that Matrigel mixed with CM from the WISP-1-shRNA group abolished neovascularization in plugs (Figure 6b) and reduced microvessel formation in the Matrigel plugs (Figure 6c). When we evaluated the level of angiogenesis by measuring hemoglobin concentrations in Matrigel plugs, we found that knockdown of WISP-1 decreases hemoglobin levels by ~40% (Figure 6d). Overall, these results indicate that WISP-1 has an important role during angiogenesis *in vivo*.

## Discussion

In recent years, much research has focused on the role of angiogenesis in osteosarcoma proliferation, development, invasion, and metastasis.^34^ VEGF-A has been recognized as being a major contributor to angiogenesis.^35^ In this study, we found that WISP-1 affects VEGF-A production in human osteosarcoma. Specifically, WISP-1 increases VEGF-A expression and angiogenesis through the FAK/JNK/HIF-1*α* pathways and down-regulates miR-381 expression.

The matricellular protein WISP-1 is a member of the CCN protein family.^36^ In human tissue, WISP-1 is detected in several organs, such as the lung, pancreas, heart, ovary, kidney, placenta, small intestine, and spleen. In adulthood, WISP-1 expression has been observed in the developing skeleton.^37^ Much evidence has determined that WISP-1 has a key role in tumor cell proliferation, migration, and survival *in vitro*, as well as tumor growth and metastasis *in vivo.*^11^ Our previous research indicated that WISP-1 promotes oral squamous cell carcinoma angiogenesis through VEGF-A expression.^28^ Here, we found that WISP-1 was up-regulated in human osteosarcoma cells. Furthermore, WISP-1 expression was positively correlated with VEGF-A in human osteosarcoma cells. Our results suggest that WISP-1 may be a novel target in the metastasis and angiogenesis of osteosarcoma.

Evidence up until now has suggested that WISP-1 has an important role in tumorigenesis.^38^ However, the mechanism by which WISP-1 modulates cellular function and which receptors it uses to transmit signals has remained unclear.^11^ Different integrins have been identified as functional receptors for CCN proteins, including WISP-1.^39^ Previous studies have shown that in sepsis-induced lung injury, WISP-1-*α*v*β*3 integrin signaling is involved in TLR4 pathways in macrophages, and may be an important contributor to TLR4/CD14-mediated inflammation.^40^ Moderate tidal volume mechanical ventilation (MTV) exaggerates Poly(I:C)-induced lung injury, in a WISP-1-integrin *β*3-dependent manner, involving the activation of the ERK pathway. Therefore, the WISP-1-integrin *β*3 pathway may be an important target for lung injury therapy.^41^ In cultured vascular smooth muscle cells (VSMCs), WISP-1 stimulates the adhesion and migration of VSMCs in a dose-dependent manner through integrin *α*5*β*1.^42^ WISP-1 also positively influences osteogenesis by enhancing the effects of BMP-2 through binding to integrin *α*5*β*1.^43^ In this study, our findings indicate that WISP-1 promotes VEGF-A expression and angiogenesis through integrin *α*v*β*3, which is consistent with our previous study, in which WISP-1 was found to increase cell motility and matrix metalloproteinase expression via the integrin *α*v*β*3-dependent pathway.^15^ These results demonstrate a pivotal role of integrin *α*v*β*3 in WISP-1-mediated tumor angiogenesis and metastasis.

It is well known that phosphorylated FAK is essential for the VEGF-A signaling pathways and angiogenesis.^30^ Previous research has reported that cartilage oligomeric matrix protein (COMP)-Ang1 induces an interaction between Tie2 (an Ang1 receptor) and FAK, then increases pp38, pSAPK/JNK, and pERK-mediated MAPK activation, finally leading to EPC migration.^44^ In nasopharyngeal carcinomas, JNK is activated by FAK through the Akt signaling pathway.^32^ Isothiocyanates inhibit the migration and invasion of C6 glioma cells via the FAK/JNK pathway.^31^ Thus, FAK regulates JNK activation through activation of the Tie2-FAK and Akt pathways. In this study, we found that JNK phosphorylation was reduced by FAK inhibitor. In contrast, the JNK inhibitor had no effect on FAK phosphorylation. This study showed that JNK is a downstream target of FAK in WISP-1-induced tube formation and migration of EPCs.

HIF-1*α* is highly expressed in hypoxic conditions and degraded in normoxic conditions.^45^ VEGF-A is considered to be a master regulator of angiogenesis.^46^ Hypoxia is one of the main regulators of VEGF-A expression, as it is a direct transcriptional target of HIF-1*α.*^47^ In our study, we found that pretreating cells with a HIF i or siRNA reduced WISP-1-promoted VEGF-A expression and angiogenesis. Previous research has shown that the HIF-1*α* pathway is regulated by activation of FAK.^48, 49, 50^ Here, we explored the effects of FAK or JNK inhibitors on HIF-1*α* and VEGF-A protein expression, as well as WISP-1-induced HRE-luciferase activity. Our data suggest that the FAK/JNK/HIF-1*α* signaling pathways have an important role in WISP-1-induced angiogenesis in human osteosarcoma cells.

MiRNAs are short noncoding RNAs that function as negative regulators of gene expression,^16^ which is important for many fields of homeostasis and disease, including cancer development.^17^ MiR-381 has been reported to be dysregulated in several human cancers.^22^ However, the function and mechanism of miR-381 in human osteosarcoma remains unclear. In the present study, we used open-source software (miRWalk, TargetScan, and microrna.org) in an attempt to determine which miRs regulate the expression of VEGF-A, and to rank the top 14 miRNAs harboring VEGF-A binding sites, including miR-381. Here, we found that miR-381 mimic decreased VEGF-A expression in MG-63 cells and elevated levels induced by WISP-1, but had minimal effect upon WISP-1-induced EPC tube formation and migration. Previous research has shown that WISP-1 is an angiogenesis-related gene,^51^ which implies that more angiogenesis-related proteins are yet to be discovered that will be stimulated by WISP-1.

In primary osteosarcoma tissue, the expression of miR-381 is negatively correlated with VEGF-A or WISP-1. Co-transfection with miR-381 mimic reduced WISP-1-induced VEGF-A expression as well as EPC tube formation and migration. Our results highlight the significance of miR-381 in WISP-1-induced angiogenesis, and suggest that restoration of miR-381 may be a potential therapeutic strategy for osteosarcoma angiogenesis. A search of the microrna.org database identified that FAK protein is associated with the miR-381 binding site, but there are no published reports illustrating this. Whether miR-381 also regulates WISP-1-mediated VEGF-A expression through down-regulation of the FAK pathway requires investigation in future studies.

In conclusion, WISP-1 enhances VEGF-A expression in osteosarcoma and promotes EPC angiogenesis through integrin *α*v*β*3 and the FAK/JNK/HIF-1*α* pathways, as well as via down-regulation of miR-381 expression. Thus, WISP-1 may be serve as a new molecular therapeutic target in osteosarcoma angiogenesis.

## Materials and Methods

### Materials

Recombinant human WISP-1 and VEGF-A were purchased from PeproTech (Rocky Hill, NJ, USA). *α*-Tubulin, JNK, FAK, HIF-1*α*, WISP-1, VEGF-A, p-JNK, and p-FAK antibodies were purchased from Santa Cruz Biotechnology (Santa Cruz, CA, USA). SP600125, HIF i, and FAK i were purchased from Calbiochem (San Diego, CA, USA). Smartpool ON-TARGET siRNAs of control, JNK, FAK, and HIF-1*α* were purchased from Dharmacon Research (Lafayette, CO, USA). The MiRNome microRNA Profilers QuantiMir kit was purchased from System Biosciences (Mountain View, CA, USA). Mimics of control and miR-381 were purchased from Invitrogen (Carlsbad, CA, USA). All other chemicals were purchased from Sigma-Aldrich (St. Louis, MO, USA).

### Cell culture

The osteosarcoma cell line used in this study was MG-63, purchased from the Bioresource Collection and Research Center (BCRC; Hsinchu, Taiwan). The cell line was subjected to Short Tandem Repeat analysis at the BCRC before commencement of our study (July 2014) to ensure quality and integrity. The MG-63 culture medium consisted of Dulbecco's modified Eagle's medium (Gibco RL, Grand Island, NY, USA) containing 10% heat-inactivated fetal bovine serum (FBS), 20 mM HEPES, 2 mM glutamine, 100 U/ml penicillin, and 100 *μ*g/ml streptomycin. Cells were maintained in humidified air containing 5% CO_2_ at 37 °C.

Human EPCs were prepared according to previous reports.^8, 52^ The EPC culture medium consisted of endothelial basal medium-2 (EBM-2) supplemented with endothelial growth medium SingleQuots and 5% FBS (Lonza, Walkersville, MD, USA). Cells were seeded onto 1% gelatin-coated culture dishes and maintained at 37 °C in a humidified 5% CO_2_ atmosphere.

### Patients and specimens

All tissue specimens were collected from patients diagnosed with osteosarcoma who had undergone surgical resection at China Medical University Hospital. All patients gave written consent before enrollment. This study was approved by the Institutional Review Board of China Medical University Hospital.

### EPC migration assay

The EPC migration assay was performed using a 6.5 mm Transwell assay with a 8.0 *μ*m Pore Polycarbonate Membrane Insert (Coring, Coring, NY, USA) in 24-well plates. EPCs (1 × 10^4^ cells/well) were mixed with 0% EBM-2 and then seeded onto the upper chamber. The lower chambers were filled with 50% EBM-2 CM mixed with 50% osteosarcoma cell CM, then incubated for 24 h at 37 °C in 5% CO_2._ After 24 h, the cells were fixed in 4% formaldehyde solution for 15 min, washed twice with phosphate-buffered saline (PBS) and stained with 0.05% crystal for 15 min. Cells on the upper side of the filters were removed with cotton-tipped swabs and the filters were washed with PBS. Migrated cells were quantified by a manual count of stained cells.

### Tube formation assay

The tube formation assay was performed using 48-well plates. Matrigel (BD Biosciences, Bedford, MA, USA) was dissolved at 4 °C overnight, cultured in 48-well plates (150 *μ*l/well), then incubated at 37 °C for 30 min. After gel formation, EPCs (1 × 10^4^ cells) were seeded in each well and incubated with 50% MV2 CM and 50% osteosarcoma cell CM for 16 h at 37 °C. Tube formation was imaged with the inverted phase-contrast microscope. Tube branches and total tube lengths were calculated using MacBiophotonics Image J software (Bethesda, MD, USA).

### ELISA assay

Human osteosarcoma cells were cultured in 6-well plates. After reaching confluence, cells were changed to serum-free medium, then pretreated with pharmacological inhibitors for 30 min, or pre-transfected with specific siRNA, or miR-381 mimic for 24 h. After treatment, cells were incubated with WISP-1 for another 24 h. The culture medium was removed and stored at −80 °C. VEGF-A protein concentration was determined using an ELISA kit according to the manufacturer's protocol (PeproTech).

### Western blot analysis

Cellular lysates were prepared as described previously.^53^ Cellular lysate concentration was determined using a Thermo BCA Protein Assay Kit (Thermo Fisher Scientific Inc., Waltham, MA, USA). Proteins were resolved by sodium dodecyl sulfate polyacrylamide gel electrophoresis and transferred to immobilon polyvinyl difluoride membranes. The blots were blocked with 4% BSA for 1 h. After blocking, blots were incubated with primary antibodies for 1 h at room temperature, then washed by Tris-buffered saline with 0.05% Tween-20. After three washes, the blots were subsequently incubated with anti-rabbit or anti-mouse peroxidase-conjugated secondary antibody for 1 h. The blots were visualized by enhanced chemiluminescence using Kodak X-OMAT LS film (Eastman Kodak, Rochester, NY, USA). Quantitative data were obtained using a computing densitometer and ImageQuant software (Molecular Dynamics, Sunnyvale, CA, USA).

### Quantitative real-time PCR

The quantitative real-time PCR was carried out using TaqMan one-step PCR Master Mix (Applied Biosystems, CA, USA). Total RNA was extracted using a TRIzol kit (MDBio Inc., Taipei, Taiwan). The cDNA was reverse transcribed using 2 *μ*g of total RNA with an oligo(dT) primer. Each cDNA (100 ng/25*μ*l reaction) sample was mixed with sequence-specific primers and probes according to the manufacturer's instructions. Sequences for all target gene primers and probes were purchased commercially (Applied Biosystems). *β*-actin was used as the internal control. The cycling conditions consisted of 10 min of polymerase activation at 95 °C, followed by 40 cycles at 95 °C for 15 s and 60 °C for 60 s.

For miRNA detection, total RNA was extracted using a TRIzol kit (MDBio Inc., Taipei, Taiwan) and expression of miRNA was analyzed using Mir-X miRNA First-Strand Synthesis and Mir-X miRNA qRT-PCR SYBR kits (Clontech Laboratories, Inc., CA, USA). U6 snRNA was used as the internal control. The specific forward primer of miR-381 was 5′-TATACAAGGGCAAGCTCTCTGT-3′. The U6 forward and reverse primers were 5′-CTCGCTTCGGCAGCACATATACTA-3′ and 5′-ACGAATTTGCGTGTCATCCTTGCG-3′. Results were expressed as Ct values and normalized to calculate the average Ct of each sample (ΔCt), and the relative expression of miR-381 was calculated using the comparative Ct method.

### Chick chorioallantoic membrane assay

Specific pathogen-free fertilized chicken eggs were purchased from an animal health research institute (Taipei, Taiwan). The eggs were incubated in an 80% humidified atmosphere at 38 °C for 7 days. On day 7, a small window was made in the shell, and CM from control-shRNA or WISP-1-shRNA cells was mixed with Matrigel and deposited in the center of the egg. The window was resealed with adhesive tape and eggs were returned to the incubator until day 11 (*n*=10 chicken embryos per cell line). CAMs were collected for microscopic and photographic documentation. At least 10 viable embryos were tested for each treatment, and the angiogenesis effect was quantified by counting the numbers of blood vessel branches.

### *In vivo* Matrigel plug assay

Osteosarcoma cell CM was collected from control-shRNA and WISP-1-shRNA cells. Male BALB/c nude mice (4 weeks of age) were purchased from the National Laboratory Animal Center (Taipei, Taiwan). Mice were randomized into three groups: 0% DMEM (control), control-shRNA, and WISP-1-shRNA. Each group was subcutaneously injected with 0.2 ml PBS or CM mixed with 0.2 ml Matrigel. On day 10, the Matrigel plugs were excised and measured for the extent of blood vessel formation by hemoglobin assay. All animal experiments were done in accordance with a protocol approved by China Medical University's Institutional Animal Care and Use Committee (Taichung, Taiwan).

### Hemoglobin assay

Angiogenesis was analyzed using the Matrigel plug assay and hemoglobin quantification was determined with Drabkin's reagent (Sigma-Aldrich), according to the manufacturer's instructions. In brief, plugs were homogenized in 1 ml of RIPA lysis buffer and centrifuged at 1000 r.p.m. for 10 min at 4 °C. 100 *μ*l of Darkin's solution was added to 20 *μ*l of supernatant, then allowed to stand for 30 min at room temperature. Absorbance of each sample was measured at a wavelength of 540 nM by a spectrophotometer and compared with the blank as reference.

### Statistics

Data are expressed as the mean±standard error. Between-group differences were analyzed using the Student's *t*-test of variance. The difference was considered significant if the *P*-value was <0.05.
