# Supplementary material for: WISP-1 positively regulates angiogenesis by controlling VEGF-A expression in human osteosarcoma
Source: Cell Death Dis. 2017 Apr 13;8(4):e2750–. doi: 10.1038/cddis.2016.421 (PMC5477571; doi:10.1038/cddis.2016.421)
Supplement: Supplementary Information [file cddis2016421x1.docx]

**Supplemental Data**

| **miRNA name** | **Fold** | **miRNA name** | **Fold** |
| --- | --- | --- | --- |
| miR-381-3p | 0.243 | miR-299-3p | 0.596 |
| miR-185-5p | 0.316 | miR-339-5p | 0.664 |
| miR-373-3p | 0.352 | miR-452-5p | 0.687 |
| miR-134-5p | 0.432 | miR-520b | 0.852 |
| miR-205-5p | 0.464 | miR-15a-5p | 0.887 |
| miR-29b-3p | 0.523 | miR-484 | 1.101 |
| miR-34a-5p | 0.562 | miR-449a | 1.238 |

**Table S1. Representative miRNA expression after WISP-1 stimulation.**

qRT-PCR analysis of < 2-fold expression of miRNAs in MG-63/WISP-1-shRNA and MG-63/Control-shRNA cells (N=3).


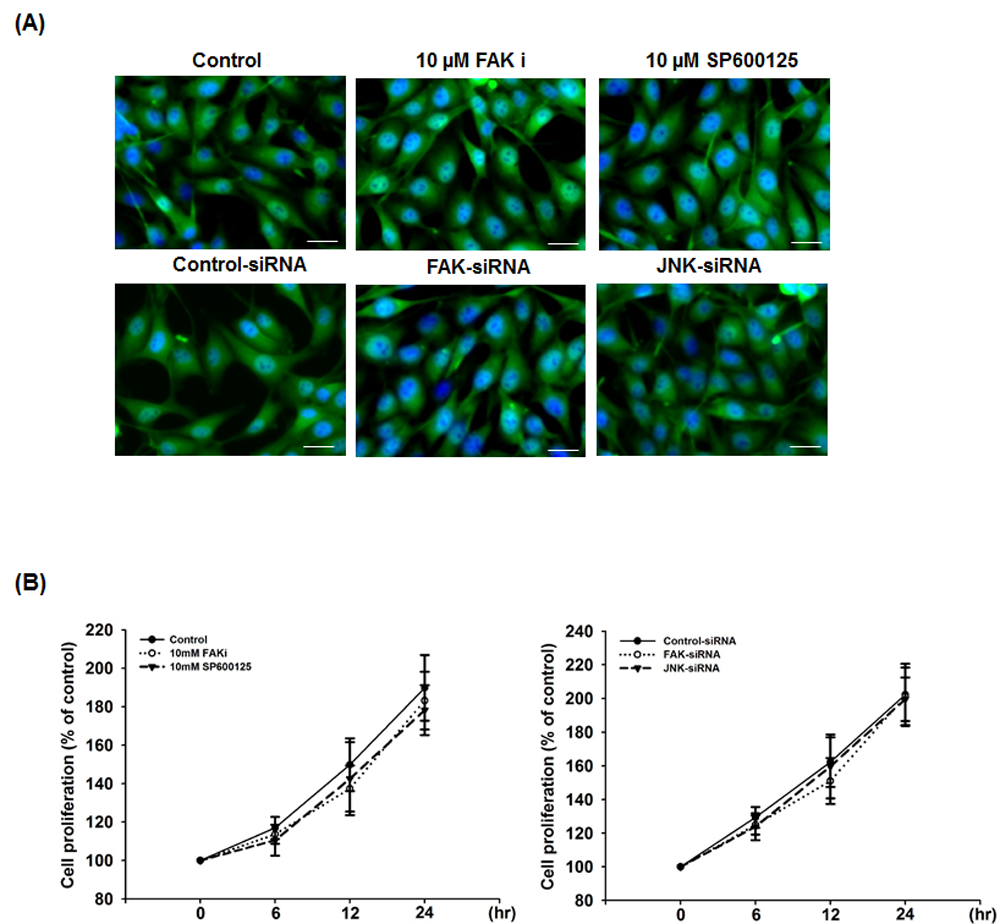


**Figure. S1 Cell viability after FAK and JNK inhibitors or siRNAs treatment.**

(A) Cells were incubated with FAK inhibitor (FAK i; 10 µM) and JNK inhibitor (SP600125; 10 µM) or transfected with siRNA for 24 h, and FITC-conjugated β-actin immunofluorescence staining was examined. The cells without any treated was used as control. (green color : β-actin, blue color : DAPI, bar=100 μm) (B) Cells were incubated with FAK inhibitor (FAK i; 10 µM) and JNK inhibitor (SP600125; 10 µM) or transfected with siRNA for 24 h, and seeded in 96-well plates (2,000 cells per well). After 0, 6, 12, 24 h, cells were collected and the viability was analyzed by using the MTT assay (N=3).


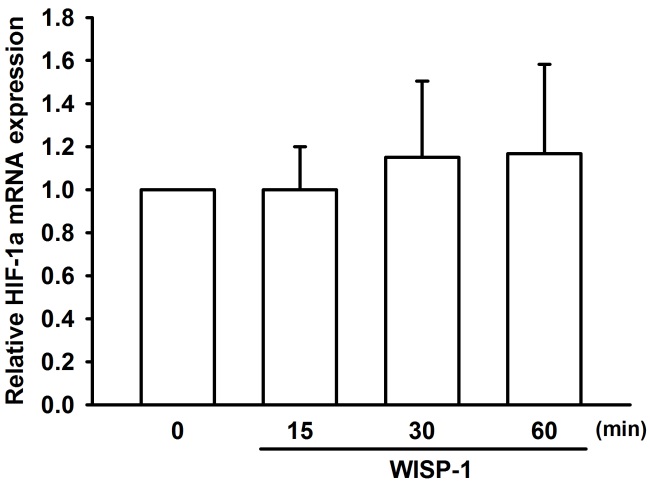


**Figure. S2 WISP-1 did not increase HIF-1α mRNA expression.**

Cells were incubated with WISP-1 (30 ng/mL) for the indicated times and HIF-1α expression was determined by RT-qPCR (N=3).


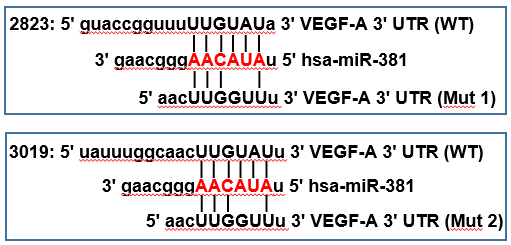


**Fig. S3 Schematic illustrations of the pmiRGLO luciferase reporter construct for examining the effect of miR-381 on VEGF-A 3’UTR.**

The full-length sequence of the VEGF-A 3’UTR is located at position 1453-3422 of VEGF-A mRNA (NM_003376). The miR-381 seed location in VEGF-A 3’UTR are 2823 and 3019. The fragment that contains the predicted miR-381 binding site was cloned into luciferase report plasmids. The construct with the VEGF-A 3’UTR mutated at the predicted miR-381 binding site are also depicted.
